# Artificial intelligence is going to transform the field of endocrinology: an overview

**DOI:** 10.3389/fendo.2025.1513929

**Published:** 2025-01-14

**Authors:** Jamal Belkhouribchia

**Affiliations:** Endocrinology Center Hasselt, AI Lab Endocrinology and Metabolism, Hasselt, Belgium

**Keywords:** artificial intelligence, AI, machine learning, endocrinology, diabetes, personalized medicine, deep learning

## 1 Introduction

The field of clinical endocrinology, as well as healthcare in general, is facing a transformative change by new technologies, especially artificial intelligence (AI). AI holds the promise to dramatically improve the way we screen, diagnose, treat, monitor, and coach patients (1, 2). Not only will AI tools make the flow of endocrine decision-making faster and more reliable, the use of AI opens the way to personalized treatment plans tailored to individual patient characteristics (3, 4).

AI is a field within computer science that encompasses machine learning (ML). ML uses mathematical algorithms designed to make predictions or classifications. These models are typically trained on known, labeled datasets and iteratively enhanced to gain the capability to make accurate predictions on unseen data (5). Deep learning (DL), a subset of ML, uses complex models that mimic the human central nervous system. DL entails the use of artificial neural networks (ANNs). ANNs consist of interconnected layers that pass information and optimize predictions by minimizing error (6). Once trained, ANNs can process vast and complex datasets to perform tasks such as predictions, classifications, and even more advanced applications like large language models (LLMs), computer vision, and multimedia generation from text inputs (7–9).

We are anticipating an unprecedented disruption of clinical endocrinology by AI. Nevertheless, most clinicians lack a proper understanding of the potential of clinical AI on the one hand, and the shortcomings and caveats on the other hand. A balanced comprehension of the underpinnings of AI is imperative to maximize its benefits. Hence, healthcare providers must familiarize themselves with this new technology but also understand its limitations. **Table 1** gives an overview of the differences between AI-based tools and conventional methods in clinical endocrinology.

**Table 1:** Comparison between AI-based tools and conventional clinical methods in endocrinology.

Aspect	AI-based tools	Conventional methods
Accuracy	Sensitivity and specificity can match or even exceed human experts (17). The accuracy depends on the quality and quantity of the data.	Performance depends on the expertise of the clinician and may vary significantly among individuals (18).
Efficiency	AI models can process large datasets quickly and provide results in real-time (19).	Diagnosis can be time-consuming, requiring multiple consultations with different specialists and manual interpretation of results.
Cost	Initial costs are high due to development and training, but operational costs decrease with scalability (19).	Lower upfront costs but higher long-term expenses due to repeated manual labor and clinician hours.
Adaptability	Capable of updating and improving as new data becomes available.	Limited by human training and reliance on established protocols.
Limitations	Performance is data-dependent; errors may arise from incomplete datasets (27). Existing biases in data can be perpetuated and even amplified in AI models, hindering their generalizability. Privacy and liability issues can also be hurdles to embrace these tools in clinical practice.	Limited by human error and cognitive biases but less dependent on computational infrastructure.

The aim of this paper is to give an overview of the potential and future direction of AI in the domain of clinical endocrinology and diabetes.

## 2 Improved risk assessment

The importance of timely risk assessment in endocrinology is well-established, and AI can significantly enhance both its speed and efficiency. For instance, Wändell and colleagues created a risk assessment tool to evaluate the risk of having *de novo* diabetes by using a stochastic gradient boosting machine learning model. Area under the curve (AUC) was between 0.773 and 0.825, indicating good discriminatory power (10). The most important risk factors were identified as being arterial hypertension and obesity. For this model, adults over 30 years old in Stokholm, Sweden were included. No information was given in relation to ethnicity. Yousef and co-workers used an interpretable ML model for the risk prediction of patients with undiagnosed type 2 diabetes mellitus. The subjects in this study were patients of a rural diabetes screening clinic in Albury, Australia. Ethnicity was not recorded. They trained two different Isolated Forest (iForest) ML algorithms. The first one on the basis of BMI (body mass index), blood glucose level, and triglycerides. The second iForest model was trained on the same parameters, supplemented with biomarkers of oxidative stress (8-isoprostane, 8-hydroxydeoxyguanosine, and oxidized glutathione), inflammation (interleukin-6, interleukin-10, interleukin-1β, and insulin-like growth factor-1), and mitochondrial dysfunction (humanin, MOTS-c, and P66Shc). The latter model outperformed the former one; F1-score increased from 0.61 to 0.81 (11). In another study, Nabrdalik et al. used a ML model for risk stratification of MASLD (metabolic dysfunction-associated steatotic liver disease) in patients with type 1 and type 2 diabetes. Patients were recruited in the diabetology ward of a hospital in Zabrze, Poland. The study initially used 80 different parameters. To determine the most discriminative predictors, feature selection was conducted using a chi-squared test. The stability of the rendered variables was assured by repeating the Monte-Carlo simulation 1,000 times. The most discriminative independent variables were employed by a multiple logistic regression model in order to predict the occurrence of MASLD (12). AI has also been employed in risk assessment of hypoglycemia in type 1 diabetes. Cichosz and colleagues developed a binary classification XGBoost (extreme gradient boost) algorithm for this aim, using CGM (continuous glucose monitoring) from 206 patients with type 1 diabetes in the United States. More than 90% was white, non-Hispanic. Their median age was 68 years. The model was validated in two independent cohorts. A total of 61,470 weeks of CGM data were included in the analysis. The XGBoost model demonstrated strong performance, with ROC-AUCs (area under the receiver operating characteristic curve) ranging from 0.81 to 0.90 across validation cohorts (13). AI also shows promise in osteoporosis risk assessment. Hong et al. argue that AI-based tools could be very beneficial in the risk assessment of patients prone to osteoporotic fractures. An individualized approach to enhance clinical management of osteoporosis is believed to be within reach with the help of cutting-edge ML models (14). This could potentially reduce morbidity and mortality, but also reduce healthcare costs and alleviate the workload of healthcare providers. Assessment of thyroid nodules can be challenging at times. Distinguishing between benign nodules and malignancy is paramount in thyroid care. Wildman-Tobriner and colleagues therefore created and validated a risk stratification system for ultrasound images of thyroid nodules, using an AI thyroid imaging reporting and data system (AI TI-RADS). A total of 378 thyroid nodules from 320 patients were included in this study. Subjects’ data was collected by using the electronic health records of Duke University Medical Center, Durham, NC, United States. No patient demographics were mentioned, besides age and sex. All nodules underwent ultrasound imaging and fine needle aspiration for cytology. Results of the AI TI-RADS were comparable to ACR TI-RADS (American College of Radiology Thyroid Imaging Reporting and Data Systems) (15). AI-driven tools are expected to improve diagnostic performance of thyroid nodules in the near future. Still challenges remain, such as inconsistent ratings by ultrasound physicians, uncertainty in cytopathological diagnosis and difficulty in discriminating follicular lesions (16). AI-driven tools hold very promising applications in risk assessment and risk prediction within the field of endocrinology and diabetes. As availability of data increases, more comprehensive models are expected to emerge.

## 3 Better and faster diagnosis

The power of AI could prove to be of great benefit in endocrine diagnostics. In order to make a diagnosis, endocrinologists rely on the clinical presentation, patient history, lab results and technical examinations, such as medical imaging. Usually a diagnosis is quite straightforward. However, occasionally doctors are confronted with challenging cases where AI might increase diagnostic speed and accuracy. Chia and co-workers, for example, employed a DL model to diagnose diabetes retinopathy (DR) in indigenous Australian patients. Data was collected at the Aboriginal Community Controlled Health Service located within a metropolitan area of Perth, Western Australia. The model outperformed a human retina specialist in terms of sensitivity; specificity was comparable (17). Joseph et al. performed a systematic review in which 34 studies were included. These studies were carried out in Asia (57%), Europe (20%), North America (12%), Australia (7%), Africa (2%) and South America (2%). Their findings indicate that performance of AI models is in fact acceptable in screening for DR. Fundus images were used for AI-driven diagnosis and compared to human graders. AI-based software in conjunction with a fundus camera can indeed facilitate the work of ophthalmologists and enhance diagnostic accuracy (18). Wu and colleagues trained a linear regression model, random forest (RF) and an XGBoost model on clinical and laboratory data of 479 patients, with diabetes mellitus, in order to diagnose diabetes neuropathy and lower limb arterial disease in patients with diabetes. Patients were recruited at Tongji Hospital, Shanghai, China. The XGBoost model proved to be more accurate in diagnosing diabetes neuropathy in comparison to the other models. Coversely, the RF model revealed to be more suitable for detecting peripheral vascular disease (19).

In some cases a timely diagnosis could be crucial for patient outcome. Here automatic AI interpretation of lab results or other patient data could provoke an alarm, so the physician can swiftly attend to the patient. For instance, Tirado-Aguilar et al. underscored the importance of a timely diagnosis in gestational diabetes in order to avoid adverse neonatal and maternal outcomes (20).

AI-based diagnostic tools are believed to become as normal in daily medical practice as the use of a stethoscope is today. We are bound to see a staggering progression in the field of diagnostic AI in endocrinology and diabetes in the years to come.

## 4 Personalized treatments

One of the most promising opportunities of AI in endocrinology is the possibility to forge a treatment on the basis of individual patient distinctions. Personalized medicine is thus coming within reach. In the years and decades to come, we will leave the one-size-fits-all approach and shift towards optimized therapies with the highest efficiency while limiting adverse effects. Long and co-workers developed ML models, trained on 9 different biomarkers to predict responders versus non-responders to metformin in type 2 diabetes patients. Data was collected at the Beijing Friendship Hospital, Capital Medical University, Beijing, China. The F1 scores of the XGBoost, KNN (K-Nearest Neighbors), NB (Naive Bayes), RF and SVM (Support Vector Machine) models were 0.830, 0.517, 0.898, 0.864 and 0.475, respectively (21). This strategy could be expanded to other drugs in order to compose the best possible treatment for each individual patient. Popova and co-workers are performing a trial with an AI-driven app in order to help women with gestational diabetes mellitus to control their glycemic levels. Patients are recruited from the outpatient department of the Perinatal Center of the Almazov National Medical Research Center, and from antenatal clinics, all located in Saint Petersburg, Russia. Prognoses of their glucose level 1 hour postprandial are given every time they input their meal data (22). This way patients could anticipate and adjust their current meal in a timely manner to avoid hyperglycemia. Closed loop artificial pancreas systems are a new and exciting technology that can greatly increase the quality of life for type 1 diabetes patients. Several AI algorithms are already being developed in this field (23). AI-based tools can also be very helpful in thyroid pathologies by assisting doctors to optimize thyroid test prescriptions, to guide them in test interpretation, and in clinical decision-making (24). Personalized medicine is believed to become mainstream once AI-tools are sufficiently developed and accepted.

## 5 Monitoring patients from a distance

The healthcare system is too overloaded, demographics are going to make this problem even worse in the future; AI-enhanced distance monitoring can mitigate this problem. A part of the solution may lie in the use of wearable devices and sensors in order to monitor patients without the need of direct oversight by a medical professional. For instance, Promphet et al. introduced a wearable sensing device in conjunction with a smartphone app in order to monitor blood glucose levels by applying an XGBoost regressor machine learning model. Subject demographics were not mentioned in this study. This device could indeed empower patients to take control of their glucose levels without the need of a doctor (25). ML models were trained on diverse patient data by Juyal and colleagues in order to detect subtle risk patterns in real time that can facilitate arterial hypertension (26). Besides this kind of sophisticated technologies, we see an increasing use of wearables nowadays. These could provide interesting data for the development of monitoring algorithms in the forthcoming years. AI could also help in distinguishing patients that need immediate attention, and those that can withstand a delay. However interesting wearable technologies are, some problems remain to be solved. Privacy concerns necessitate a high level of encryption. And the cost of such wearable devices has also been subject of discussion. It is conceivable, however, that cost savings would offset investment costs in the long run.

## 6 Ethical considerations and limitations of AI

Despite the enormous potential of AI in endocrinology, there are some limitations and caveats that could hinder widespread adoption of this exciting new technology. The first limitation is the fact that models are dependent on the quality of the data they’re trained on. Missing data, incorrectly labeled data, errors, and mistakes can give rise to inaccurate models (27). The second constraint lies in understanding that generalizability can be problematic as models might use data of certain ethnic groups, geographical locations, social strata, gender, or age category, and apply them to patients that are not aligned with the training data the model operates on (28). The third hurdle entails privacy concerns regarding the use of sensitive patient data (29). Certain regulations have to be adhered to. This can complicate and inhibit the application of AI models in some situations. The last impediment relates to the issue of liability. It remains unclear whether the doctor using AI-based tools is responsible for patient outcomes, or if the company providing the application holds the final responsibility (30). There are still quite some ethical questions to be answered before AI can be fully embraced in clinical endocrinology.

## 7 Conclusions

In conclusion, AI has the potential to revolutionize clinical endocrinology by enhancing risk assessment and diagnosis, offering personalized treatments, and allowing remote monitoring. However, for this transformation to be fully realized, healthcare professionals must proactively embrace AI, understanding both its benefits and limitations. Without adequate preparation and comprehension, the field of endocrinology may miss a pivotal opportunity to improve patient care through this groundbreaking technology. It is essential for clinicians to engage with AI responsibly, ensuring they are equipped to navigate both its promises and its ethical and practical challenges.
